# Ketogenic diet for human diseases: the underlying mechanisms and potential for clinical implementations

**DOI:** 10.1038/s41392-021-00831-w

**Published:** 2022-01-17

**Authors:** Huiyuan Zhu, Dexi Bi, Youhua Zhang, Cheng Kong, Jiahao Du, Xiawei Wu, Qing Wei, Huanlong Qin

**Affiliations:** 1 Department of Pathology, Shanghai Tenth People’s Hospital, Tongji University School of Medicine, Shanghai, China; 2 Research Institute of Intestinal Diseases, Tongji University School of Medicine, Shanghai, China; 3 Department of Gastrointestinal Surgery, Shanghai Tenth People’s Hospital, Tongji University School of Medicine, Shanghai, China; 4 Shanghai Clinical College, Anhui Medical University, Hefei, China

**Keywords:** Health policy, Metabolic disorders

## Abstract

The ketogenic diet (KD) is a high-fat, adequate-protein, and very-low-carbohydrate diet regimen that mimics the metabolism of the fasting state to induce the production of ketone bodies. The KD has long been established as a remarkably successful dietary approach for the treatment of intractable epilepsy and has increasingly garnered research attention rapidly in the past decade, subject to emerging evidence of the promising therapeutic potential of the KD for various diseases, besides epilepsy, from obesity to malignancies. In this review, we summarize the experimental and/or clinical evidence of the efficacy and safety of the KD in different diseases, and discuss the possible mechanisms of action based on recent advances in understanding the influence of the KD at the cellular and molecular levels. We emphasize that the KD may function through multiple mechanisms, which remain to be further elucidated. The challenges and future directions for the clinical implementation of the KD in the treatment of a spectrum of diseases have been discussed. We suggest that, with encouraging evidence of therapeutic effects and increasing insights into the mechanisms of action, randomized controlled trials should be conducted to elucidate a foundation for the clinical use of the KD.

## Introduction

“Diseases enter by the mouth”, the literal meaning of the Chinese idiom “” (bìng cóng kǒu rù), which was first recorded in the Tsin/Jin () Dynasty, was initially assumed to convey the concept of dietetic hygiene; however, at present, the idiom aptly emphasizes the fact that dietary factors are closely associated with many diseases.^1^ Dietary planning is increasingly popular, not only as an intervention to maintain health but also as an important non-pharmaceutical option for fighting disease. Choosing a proper diet can have profound implications for health and may induce therapeutic effects. At present, there are numerous types of diets, including low-carbohydrate diets (LCDs; e.g., ketogenic diet [KD]), paleo-type diets, plant-forward diets, intermittent fasting, clean eating, traditional regional diets (e.g., Mediterranean diet), and other specifically designed diets (e.g., dietary approaches to stop hypertension diet, Mayo Clinic diet), that diversify food patterns or fulfill specific purposes. With the explosion of experimental and clinical research on microbiota in the past decade, the important roles of microbiota in health and disease have become well-known.^2^ Recently, microbiota-directed food invention, which was developed to exert therapeutic effects through the manipulation of gut microbiota components, was found to be an effective dietary supplementation strategy for undernourished children.^3,4^ One of the diets on the burgeoning list of diets, the KD, has a long history of clinical use and has recently gained considerable interest owing to its promising potential effects on a wide spectrum of diseases.

**Fig. 1:**
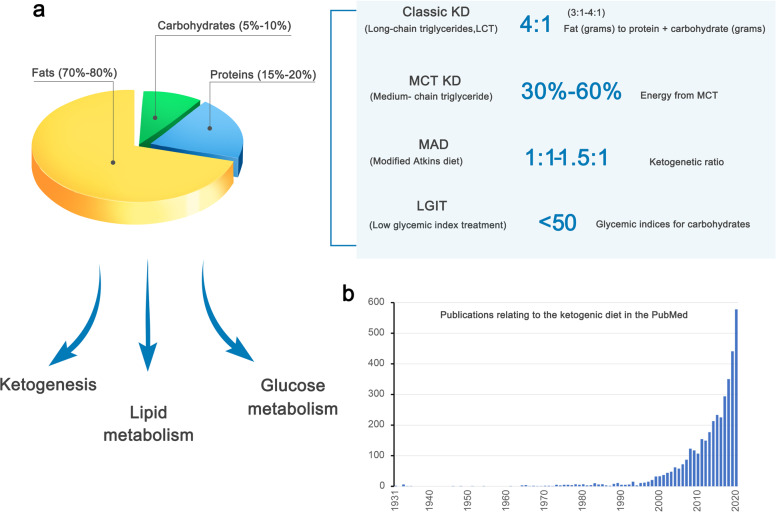
The composition and metabolic effects of the ketogenic diet, which have increasingly generated interest. **a** The compositional features of the classic KD and its variants are shown. **b** The number of publications obtained for the search term “ketogenic diet” in PubMed is shown by the year of publication. Articles published before 1931 were not included due to the unavailability of PubMed records predating this timepoint

The KD comprises a high-fat component, very low carbohydrates, and adequate proteins (Fig. 1),^5–7^ and has been clinically used since the early 1920s to control seizures in patients with epilepsy, especially those who do not respond adequately to antiepileptic medication.^7–9^ The history of dietary interventions used as “cures” for epilepsy possibly dates back to 500 before christ, whereas fasting has been recognized as an effective therapy against epilepsy and has even been recorded in the Hippocratic collection.^8^ Modern implementation of fasting as an antiepileptic treatment began in 1911,^8^ when it was noted that a diet containing few carbohydrates but a high proportion of fat could produce acetone and beta-hydroxybutyric acid (β-HB), similar to what is seen with starvation,^10^ and that alternative ketonemia-producing approaches might achieve effects similar to that of fasting.^5^ In 1921, Russel Wilder first proposed that a ketone-producing diet could be as effective as fasting for the treatment of epilepsy, and coined the term “ketogenic diet”.^8^ In particular, the KD can mimic the metabolic effects of fasting without significant calorie deprivation. The KD enjoyed wide popularity as a medical approach for treating epilepsy for nearly a decade before the introduction of antiepileptic agents, such as diphenylhydantoin.^8^ The KD re-emerged in the 1990s and became well established as an option for drug-resistant epilepsy.^8,9,11,12^ In the past few decades (Fig. 1), the KD has received extensive interest because of its beneficial effects in a number of diseases, such as neurological disorders, obesity, type 2 diabetes mellitus (T2DM), cancer, intestinal disorders, and respiratory compromise.^13–23^ Here, we provide a comprehensive review of the KD, covering the therapeutic effects, relevant mechanisms, and clinical evidence underlying the implementation of the KD in various diseases.

## Types of the ketogenic diet

The KD is characterized as a high-fat, very-low-carbohydrate diet. Several variant KD that show similar efficacy to that of the original form has been developed to date, and offer flexibility to increase compliance with the regimens.^24,25^ There are four major types of the KD with proven efficacy: the classic long-chain triglyceride (LCT) KD, medium-chain triglyceride (MCT) KD, modified Atkins diet (MAD), and low glycemic index treatment (Fig. 1).^24^

The classic LCT KD is the most traditional type of the KD, is widely used in the clinical setting, and incorporates a 4:1 ratio of fat (in grams) to protein plus carbohydrate (in grams).^26,27^ Fat provides 90% calories, and its predominant source is food-derived LCT, and a 3:1 or lower ratio may be used.^24^ Moreover, the low ratios are appropriate for the KD initiation in infants, whereas in older children, initiation with a 4:1 ratio, followed by a reduced ratio may be more effective.^24,28^ Furthermore, there is evidence that calorie and fluid restriction is unnecessary as no beneficial effect was proved with these two factors.^24,29^

Due to the severe carbohydrate restriction, the LCT KD is unpalatable, difficult to prepare, and, therefore, difficult to maintain.^30^ In 1971, the MCT (C6-C12) KD was devised.^30^ The dietary use of MCT oil is more acceptable and is more ketogenic than LCTs.^30–33^ The MCT KD has better flexibility in diet ratios than the LCT KD, and the calorie intake is calculated based on the percentage of energy derived from MCT.^24,31^ In addition, there is clinical evidence of the equivalent efficacy of the MCT and LCT KD.^12,32^ However, the MCT KD is frequently associated with gastrointestinal side effects.^24,31^

The MAD is based on the Atkins diet, which was popularly used in weight loss^34–36^ and shares similar food choices with the classic KD, but without the need for precise weighing of ingredients. The MAD does not have a strict ketogenic ratio, which typically ranges from 1:1 to 1.5:1 and, sometimes, can reach 4:1.^35^ Moreover, the MAD does not include protein, fluid, or calorie restrictions. Carbohydrate intake in the MAD is restricted to 10–15 g/day in the first month and can be subsequently increased to 20 g/day.^37,38^ There is clinical evidence supporting the efficacy of the MAD in children with intractable epilepsy.^37–44^

The low glycemic index treatment is based on the concept that the protective effect of the KD relies on stable glucose levels,^45^ but has a liberalized regimen with low-carbohydrate composition to minimize glycemic increases (glycemic indices <50),^45^ and is an effective antiepileptic intervention in children with intractable epilepsy.^44–49^

Despite the abovementioned evidence that suggests the similar efficacy of the four types of KD, it is unclear whether the mechanisms of action of these diets differ.

## The impact of the ketogenic diet on metabolism

### Lipid metabolism

The metabolism of blood lipids during KD is often a concern. In the presence of oxygen, most cellular energy originates, through glycolysis, from glucose-metabolized pyruvate, which then undergoes oxidative phosphorylation within mitochondria. In the absence of glucose, cellular energy is produced by the degradation of fatty acids.^50^ A low-carbohydrate, high-protein, and high-fat diet can be unhealthy as it may lead to an increase in the circulating low-density lipoprotein (LDL), cholesterol, and triglyceride (TG) concentrations. As for liver fat metabolism, from the perspective of diet metabolism, a low total and saturated fat/high-carbohydrate diet can effectively manage liver fat storage by limiting exogenous fats.^51^ However, the KD has potential health benefits with regard to these cardiovascular risk factors, and recent animal and clinical studies provided ample evidence that cutting carbs can actually lower total cholesterol, increase high-density lipoprotein (HDL), and reduce blood TG levels.^52,53^ With the premise of ensuring constant total calorie intake, the KD reduces carbohydrate intake, lowers serum insulin levels, increases insulin sensitivity, and enhances fat catabolism, thus reducing blood lipids.^14^ Due to increased de novo lipogenesis and decreased fatty acid oxidation and/or ketone production, higher carbohydrate intake may be detrimental to the net loss of liver fat. In contrast, low-carbohydrate/high-fat KD significantly increases the rate of whole-body fatty acid oxidation and liver ketogenesis.^54,55^ Therefore, KD has been shown to reduce liver fat.^56,57^ Moreover, the KD induces the expression of fibroblast growth factor-1 and promotes the hepatic clearance of TGs.^58^ In addition, the KD can increase the size and volume of LDL-C particles,^59^ which is believed to reduce the risk of cardiovascular disease, as smaller LDL particles have higher atherogenic activity. Furthermore, the KD affects endogenous cholesterol synthesis. β-Hydroxy β-methylglutaryl-CoA reductase, a key enzyme in cholesterol biosynthesis, is activated by insulin. Therefore, increased blood glucose concentrations and higher insulin levels lead to increased endogenous cholesterol synthesis. Thus, reducing dietary carbohydrates and proper cholesterol intake will lead to the inhibition of cholesterol biosynthesis.

### Glucose metabolism

There are two sources of glucose in humans: glycogenic amino acids and glycerol that are released by TG lysis.^60,61^ The importance of the latter source increases during ketosis. In the first few days of the KD, glycogenesis from amino acids is the main source of glucose. Subsequently, the contribution of amino acids is reduced, whereas the amount of glucose obtained from glycerol increases. In fact, TG-hydrolysis-induced glycerol can generate more than 16% glucose in the liver during the KD, compared to 60% glucose after several days of complete fasting.^62^ The effect of the KD on blood sugar levels remains controversial. After fasting for several days or restricting carbohydrate intake, the glucose reserves in the body are insufficient to produce oxaloacetate in the Krebs cycle for normal fat oxidation and supply of glucose to the central nervous system.^63^ Thus, most studies believe that the KD leads to decreased blood sugar concentration and a lower insulin-to-glucagon ratio, which is beneficial for glycemic control in individuals with diabetes.^20,64^ Elevated glucagon levels are associated with hepatic glucose mobilization. A recent study analyzed the effects of KDs in exercising and sedentary rats.^65^ After 6 weeks, KD decreased insulin levels by 80%, blood sugar by 50%, TGs by 55%, and cholesterol by 20%, compared to the standard feed, whereas exercising did not bring benefits. Furthermore, a 5-year prospective study that included a total of 27,799 men and 36,875 women in Japan showed that LCDs are significantly associated with a reduced risk of type 2 diabetes in women, whereas high-fat and high-protein diets are protective factors against diabetes in Japanese women.^66^ However, Delahanty et al. arrived at the opposite conclusion. Independent of exercise and body mass index, patients with type 1 diabetes who consume high fat and LCDs have higher glycosylated hemoglobin and poorer blood sugar control.^67^ Some animal experiments have shown that glucose tolerance decreases in mice that are fed KD for 22 weeks.^68^ The KD did not prevent the decline in β-cell function, nor did it improve insulin secretion. Therefore, individual differences and treatment conditions should be considered in the clinical application of the KD.

### Ketogenic process

In the liver, excessive production of acetyl coenzyme A (acetyl-CoA) and oxidation of fatty acids leads to the production of Ketone Bodies (KBs).^69^ The acetyl-CoA molecule can be utilized in the Krebs cycle or to produce acetoacetate, which is then spontaneously converted to acetone or 3-β-hydroxybutyrate by 3-β-hydroxybutyrate dehydrogenase.^70,71^ The KBs then enter the bloodstream and can be utilized by the brain, heart, and muscle, where they produce cellular energy in mitochondria.^7,72,73^ Higher circulating KB levels lead to ketonemia and ketonuria.^74^ Under physiological conditions, the blood concentration of KBs during prolonged fasting usually is 5–7 mM, while the glucose concentration could be lowered to below 1 mM without either convulsions or any impairment of cognitive function.^75^ In diabetic ketoacidosis, the plasma KB levels can increase up to 25 mM due to insulin deficiency, with a consequent increase in the plasma glucose concentration and decreased blood pH.^74^ The KBs constitute a more efficient energy source than glucose, metabolize faster than glucose, and can bypass the glycolytic pathway by directly entering the Krebs cycle, whereas glucose needs to undergo glycolysis.^76^ Moreover, KBs cause fatty acid-mediated activation of peroxisome proliferator-activated receptor α as well as the inhibition of glycolysis and fatty acids.^77^ Therefore, KBs reduce the production of glycolytic adenosine triphosphate (ATP) and increase mitochondrial oxidation-induced ATP generation,^71^ thereby promoting mitochondrial oxidative metabolism, with resultant beneficial downstream metabolic changes.

### Ketogenic diet and gut microbiota

The effects of the KD on the gut microbiome have been reported in many murine and human studies (Table 1). Mice that were fed a 4-day KD showed significant changes in gut bacterial composition, which was characterized by an increase in *Akkermansia* and *Parabacteriodes* populations that induced an anti-seizure effect in germ-free or antibiotic-treated mice.^78^ The increased gut populations of these two bacterial genera decrease the γ-glutamyl transpeptidase level, which catalyzes the transfer of functional groups of γ-glutamyl from glutathione to an amino acid acceptor that may produce glutamate.^79^ In addition, ketogenic γ-glutatamylated amino acids decreased in the gut and in the blood, which supports the key anti-seizure effects of KD-associated microbiota.^78^ In the human gut, the post-KD production of KB by the host can partially drive gut microbial shifts, which reduces the number of intestinal Th17 cells.^19^ Similarly, using a murine model, Kong et al. demonstrated that an increase in *Akkermansia muciniphila, Lactobacillus*, and *Roseburia* following a KD plays a potential anti-colitis effect.^20^ The potential protective effects on intestinal barrier function may be related to the production of RORγt^+^CD3^-^ group 3 innate lymphoid cells and related inflammatory cytokines (IL-17α, IL-18, IL-22, CCL-4).^20^ Another study of a 16-week KD revealed beneficial effects of the ketogenic-induced microbiota, including improved neurovascular functions in mice and reduced risk of Alzheimer’s disease.^80^ These beneficial effects may be related to changes in the gut microbiota composition, including an increase in the beneficial bacteria *Akkermansia muciniphila* and *Lactobacillus*, which produce short-chain fatty acids. Interestingly, Ma et al. also found a decrease in the numbers of pro-inflammatory microbes, such as *Desulfovibrio* and *Turicibacter*. Furthermore, the KD improves the gut microbiome in a murine model of autism,^81^ and Newell et al. observed an overall reduction in the microbial richness of the cecum and feces and an increased ratio of *Firmicutes* and *Bacteroides* after the administration of the KD. As carbohydrates are the basic blocks that the microbes break down to produce energy, the lower carbohydrate content in the KD results in a decline in overall microbial diversity.^82^ Furthermore, in treatment-refractory epilepsy, the KD significantly reduced the abundance of pathogenic proteobacteria (*Escherichia*, *Salmonella*, and *Vibrio*), whereas *Bacteroidetes* populations increased.^83^ Notably, *Bacteroidetes* are closely involved in the digestion and metabolism of high-fat nutrients, regulation of interleukin secretion in dendritic cells, and are associated with seizure effects in epileptic patients.^84^ Another study found differences in the gut microbiota between responders (reduced seizure frequency or seizure cessation) and non-responders (no effect on seizure) among children who received a KD and noted that an increase in *Bacteroides* and a decrease in *Firmicutes* and *Actinomycetes* populations in the responders.^85^ On the other hand, populations of *Clostridia*, *Ruminococcus*, and *Lachnospiraceae* (*Firmicutes* phylum) increased in non-responders. These data suggest that the KD-induced gut microbiota changes should be considered as a potential biomarker for the efficacy of antiepileptic therapy. Moreover, an updated study showed that KD potentiates cognitive impairment induced by intermittent hypoxia in mice and increases the risk-associated *Bilophila wadsworthia.*^86^ Inhibiting Th1 cell development abrogates the adverse effects of both *B. wadsworthia* and environmental risk factors on cognitive impairment.^86^ Taken together, these findings identify the potential select gut bacteria that contribute to KD effects on target site in mice and humans.

**Table 1:** Summary of the gut microbiota changes induced by the ketogenic diet

Year	Model	Treatment	Increase	Decrease
Olson et al.^78^	Mice	4-day KD	*Akkermansia*, *Parabacteriodes, Sutterella, Erysipelotrichaceae*	*Allobaculum, Bifidobacterium, Desulfovibrio*
Kong et al.^20^	Mice	16-week KD	*Akkermansia muciniphila*, *Lactobacillus, Roseburia*	*Desulfovibrio*, *Turicibacter*
Xie et al.^83^	Epileptic and healthy infants	/	*Bacteroidetes*	*Escherichia*, *Salmonella*, *Vibrio*
Zhang et al.^85^	Seizure patients	6-month KD	*Bacteroides, Clostridiales, Ruminococcaceae, Rikenellaceae, Lachnospiraceae, Alistipes*	*Firmicutes, Actinomycetes*
Olson et al.^86^	Mice	7-day KD	*Bilophila wadsworthia*	*Clostridium cocleatum*

**Fig. 2:**
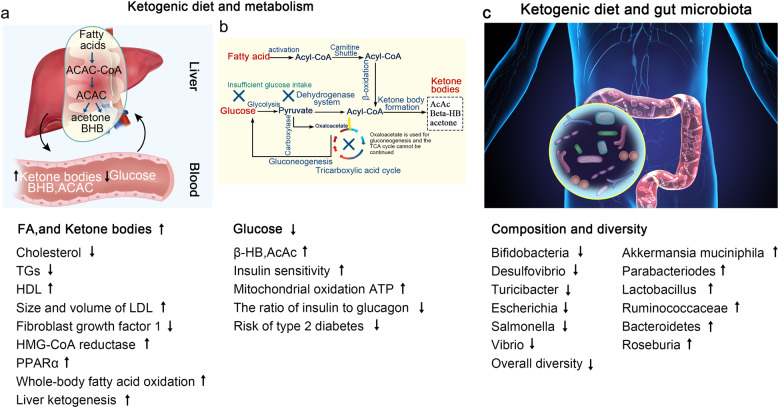
Summary of KD-induced changes in metabolism and gut microbiota. **a**, **b** The KD increases the levels of FA and KBs and decreases plasma glucose concentrations through different pathways. **c** The KD alters the composition and diversity of microbiota as follows: the increased abundance of *Akkermansia muciniphila*, *Parabacteriodes*, *Lactobacillus*, *Ruminococcaceae*, *Bacteroidetes*, and *Roseburia*, and reduced populations of *Bifidobacteria*, *Desulfovibrio*, *Turicibacter*, *Escherichia*, *Salmonella*, and *Vibrio*

In the potential physiological application of the ketogenic diet, some studies have found that KD could extend longevity and reduce midlife mortality in the mouse model.^87,88^ In fact, the mechanism of how KD works in our body from the intestine to the target site is still controversial. Based on the gut microbiota, the ketone body itself can selectively inhibit the growth of *bifidobacteria*, thereby reducing the level of intestinal pro-inflammatory Th17 cells.^19^ The ketone bodies are also involved in multiple metabolic pathways, and protective effects of ketone bodies may lead to improvement in health status and delay both aging and the development of related diseases through improving mitochondrial function, antioxidant and anti-inflammatory effects, histone and non-histone acetylation, β-hydroxybutyrylation of histones, modulation of neurotransmitter systems and RNA functions.^89^ Thus, the accumulation of ketone bodies can at least partly explain the influence of the gut microbiota by KD,^20^ which thereby inhibiting colitis, improving several diseases such as epilepsy. The summary of changes in metabolism and gut microbiota induced by the ketogenic diet is shown in Fig. 2.

## Function in endocrine and metabolic disorders

### Type 2 diabetes mellitus

T2DM is characterized by chronic hyperglycemia with fasting plasma glucose concentrations ≥126 mg/dL and glycated hemoglobin (HbA1c) ≥6.5%.^90^ As dietary carbohydrates are the major macronutrients that increase glycemic levels,^91^ it is logical to reduce the dietary carbohydrate intake to treat T2DM. Researchers have found that carbohydrate restriction has the greatest effect on reducing postprandial and overall glucose concentrations and the HbA1c.^92–96^ For example, in a study comparing the effect of a very-low-carbohydrate ketogenic diet (VLCKD) and a low-calorie diet on blood glucose levels in diabetic patients, the decrease in blood glucose concentrations was greater in the VLCKD group than in the low-calorie diet group for 24 weeks. More importantly, the blood glucose level of the VLCKD group was ~1 mM lower than that of the low-calorie diet group and reverted to a normal level after 24 weeks. However, the blood glucose level of the low-calorie group leveled out at 16 weeks and remained elevated thereafter. At 24 weeks, the HbA1c level in the VLCKD group decreased to 6.2%, compared to >7.5% in the low-calorie diet group.^96^ Similarly, a meta-analysis revealed that VLCKD resulted in a significant decrease in HbA1c and weight loss after 3 months and after 6 months, however, it was not better after 12 months compared to a control diet.^97^ VLCKD showed more beneficial effects on serum triglycerides and high-density lipoprotein cholesterol levels and reducing antidiabetic medications for up to 12 months.^97^

**Fig. 3:**
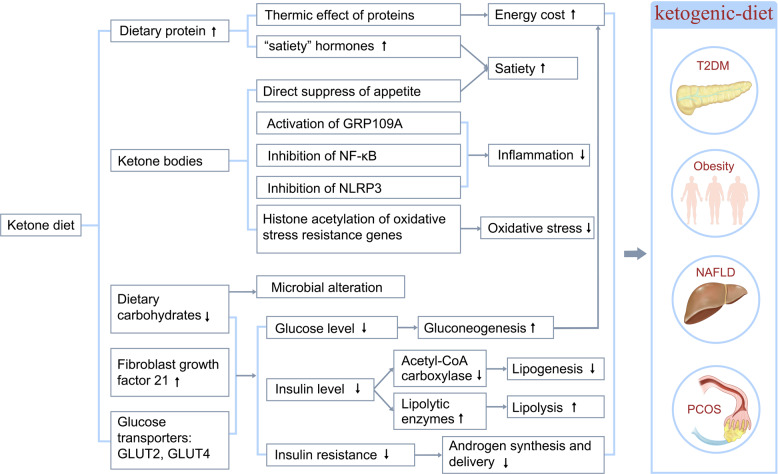
Possible mechanisms whereby the ketogenic diet ameliorates metabolic disorders. The mechanisms, through which the ketogenic diet ameliorates endocrine and metabolic disorders, including T2DM, obesity, NAFLD, and PCOS, are shown. Ketogenic diets exert therapeutic effects on metabolic disorders through various mechanisms, including reduction of plasma glucose, glycated hemoglobin levels, and serum insulin levels; improvement of insulin sensitivity; increased satiety; and decreased inflammation

Hyperglycemia is the most frequent characteristic of T2DM; however, the pathophysiology of T2DM involves insulin resistance and hyperinsulinemia. Therefore, reducing insulin levels should be a therapeutic target in the treatment of T2DM. The homeostatic model assessment of insulin resistance is an indicator for evaluating insulin resistance. The consumption of the KD decreased the homeostatic model assessment of insulin resistance in patients with T2DM from −0.4 to −3.4.^98–101^ A systematic meta-analysis that included 13 studies showed that the KD not only guarantees the basic supply of nutrients but also maintains a negative balance of energy, thereby decreasing the fluctuation and reduction of insulin secretion caused by reduced carbohydrate intake as well, which eventually leads to increased insulin sensitivity.^102^ Thus, KD improves glycemic control in T2DM patients by reducing glucose uptake and improving systemic insulin sensitivity (Fig. 3).

The molecular mechanism underlying the KD-induced improvement of T2DM clinical outcomes has been investigated in both the system biology approach and mouse model studies. Using a cell network-analysis approach, researchers identified a strong correlation between insulin resistance and the main pathways of ketosis. Glucose transporter type 4, an effector protein of the insulin-resistance pathway, directly correlates with proteins, such as Hydroxyacy1-CoA dehydrogenase 1 and Acyl-coenzyme A oxidase 1, that are involved in the KD-induced pathways.^103^ In ob/ob mice studies, several molecules are involved in the improvement of hyperglycemia and hyperinsulinemia during the KD. The expression of certain O-GlcNAc-modified proteins is altered when the KD improves hyperglycemia.^104^ Fatty acid synthase and acetyl-CoA carboxylase 1, which are two key enzymes that are involved in hepatic lipogenesis, are present in regular diet fed-ob/ob mice but absent in the KD-fed mice.^105^ KD administration decreased liver mRNA expression of Glucose transporter type 2 while increased that of Fibroblast growth factor 21 in diabetic mice.^106^ Glucose transporter type 2 plays an important role in glucose induced-insulin secretion in pancreatic β cells^107^ thus decreased Glucose transporter type 2 expression suggests a decreased insulin level and improved insulin resistance in T2DM. Fibroblast growth factor 21 is an important target gene of peroxisome proliferator-activated receptor α, which promotes lipid catabolism and improves insulin resistance.^108^ Moreover, the β-HB could inhibit nuclear factor κB (NF-κB) signaling,^109^ an upregulated inflammatory pathway associated with the pathogenesis of T2DM.^110^

Caution should be exercised when prescribing the KD to T2DM patients on other drug treatments, such as sodium glucose cotransporter 2 (SGLT2) inhibitors and insulin. SGLT2 inhibitors, which confer cardiovascular benefits in T2DM patients, can also exert pro-ketogenic effects by mediating a metabolic switch from glucose to lipid utilization. Thus, T2DM patients who are already receiving SGLT2 inhibitors will have a significantly higher risk of developing euglycemic diabetic ketoacidosis if placed on the KD; therefore, the KD should not be prescribed to T2DM patients receiving SGLT2 inhibitors.^111^ Carbohydrate restriction may increase the risk of hypoglycemia in patients receiving insulin and insulin secretagogues; thus, it is recommended that the drug dosage should be modified based on the goal of glycemic control and the type of antidiabetes therapy when prescribing the KD to T2DM patients.^112^

### Obesity

With the increasing prevalence of obesity, the 21st century has witnessed the emergence of various diet programs, with the KD at the forefront, for promoting weight loss and enhancing physical performance. Many studies have demonstrated that the KD is a potentially promising diet for reducing obesity while maintaining the capacity for physical activity. A study conducted in 2016 showed that short-term KD followed by an almost carbohydrate-free diet effectively reduced body weight, waist circumference, blood pressure, and insulin resistance in clinically healthy morbidly obese adults with body mass index (BMI) ≥ 45 kg/m.^2,113^ In a long-term study, the KD significantly decreased BMI, blood cholesterol, and plasma glucose, and increased weight loss, thereby reducing the risk factors for various obesity-associated chronic diseases, in obese hypercholesterolemic patients with BMI > 35 kg/m^2^ without any side effects.^114^ In a controlled study enrolling 20 participants who received the VLCKD, a significant improvement in biochemical parameters was observed after 8 weeks of KD adherence, which included a reduction in BMI, LDL-C, TGs, insulinemia, and liver transaminases.

In addition, KD has a more beneficial effect on obesity than other diets. In a meta-analysis of 11 studies, significant weight reductions were reported in the LCD group when compared to the low-fat diet group. Interestingly, the authors attributed this effect to a lower energy intake rather than the macronutrient composition.^115^ In individuals assigned to a VLCKD, the body weight (weighted mean difference [WMD]−0.91 kg, 1,415 patients), TG (WMD 0.18 mmol/l, 1,258 patients), and diastolic blood pressure (WMD-1.43 mmHg, 1,298 patients) decreased, whereas HDL-C (WMD 0.09 mmol/l, 1,257 patients) and LDL-C (WMD 0.12 mmol/l, 1,255 patients) increased, and resulted in a greater weight loss than in those assigned to a low-fat diet in the long term; thus, a VLCKD may be an alternative option in obesity.^116^ Similarly, a meta-analysis of randomized controlled trials (RCTs) demonstrated that, compared to low-fat diets, KD more effectively improved the metabolic parameters associated with glycemic, weight, and lipid control in obese participants, especially those with preexisting diabetes.^117^

Plasma lipids constitute the main exigent concern with the KD in the treatment of obesity. The general opinion is that a low-carbohydrate, high-protein, and high-fat diet is potentially unhealthy because it may increase LDL-C and TGs, which is an especially important issue in obese individuals. However, several lines of evidence support the positive effects of the KD on these cardiovascular risk factors. The majority of studies amply demonstrate that reduced carbohydrate uptake can decrease total cholesterol and TG levels and increase the HDL-C level.^116,118^ Furthermore, KD increase the size and volume of LDL-C particles^59^ which could alleviate the risk of cardiovascular disease that is attributable to the higher atherogenicity of smaller LDL particles. In addition, KD influence endogenous cholesterol synthesis, whereby an increase in blood glucose and insulin levels increases endogenous cholesterol synthesis. In turn, reduced carbohydrate uptake accompanied by proper cholesterol intake inhibits cholesterol biosynthesis.

The KD is obviously effective in the weight control of obese individuals; however, the underlying mechanism is incompletely understood. Researchers have proposed several mechanisms for the KD effect on weight loss including: (1) reduced appetite due to the higher satiety effect of proteins (by increasing the concentrations of “satiety” hormones, such as glucagon-like peptide-1, cholecystokinin, and ghrelin),^119,120^ effect on appetite-control hormones,^121^ and a possible direct suppression of appetite by KBs, such as β-HB, which act both in energy/satiety signaling and in mediating the central satiety signal;^122,123^ (2) reduced lipogenesis due to improved insulin resistance^124^ and increased lipolysis due to increased expression of lipolytic enzymes, such as adipose triglyceride lipase, hormone-sensitive lipase, and lipoprotein lipase;^125^ (3) higher metabolic efficiency in consuming fats that is indicated by the reduction in the resting respiratory quotient;^126,127^ and (4) increased energy consumption due to increased gluconeogenesis, which is an energy-intensive process that costs ~400–600 Kcal/day and the thermic effect of protein, which has the highest energy cost among all the three macronutrients (Fig. 3).^128,129^

Besides fat and weight loss, the KD can exert a series of other beneficial effects on obesity. Insulin resistance is common in obese patients. Very-low-carbohydrate diets could improve glycemic control, HbA1c levels, and lipid markers in obese individuals before obvious weight loss occur, which indicates that the KD could improve metabolic markers independent of weight loss. Moreover, in isocaloric experiments, the KD showed a dramatic improvement of metabolic markers than a low-fat diet in obese participants with insulin resistance.^130^ The KD may protect against obesity-induced cognitive damage^131^ and confer positive effects on mood in obese participants.^132,133^ Another postulated beneficial effect of KD is related to longevity. Although restricted to animal studies, the KD is related to several pathways in metabolic syndrome and cancer, including increased 5′ adenosine monophosphate-activated protein kinase (AMPK) activity, inhibition of the mTOR/AKT pathway,^134^ lowering the serum ratio of insulin-like growth factor/IGF-binding protein 3^135^ and increasing peroxisome proliferator-activated receptor-γ coactivator-1α expression (a master mitochondrial metabolic regulator that can increase mitochondrial biogenesis).^136^

### Nonalcoholic fatty liver disease

Nonalcoholic fatty liver disease (NAFLD) is a highly prevalent disease that is characterized by hepatic adiposity, which comprises fatty liver, fibrosis, and inflammation. The earliest stage of NAFLD is hepatic steatosis, wherein TG accumulate in >5% of hepatocytes or the intrahepatic TG concentrations exceed 55 mg/g liver (5.5%).^137,138^ Weight loss is recommended for the general clinical management of NAFLD.

The LCDs, especially those with high-fat content, are reported to have a worsening effect on hepatic steatosis due to the influence on cholesterol levels and hepatic function. However, the beneficial effects of KD on NAFLD have been widely reported. A meta-analysis of ten studies examining the effects of LCDs on NAFLD revealed that participants with NAFLD who followed LCDs exhibited a significant reduction in intrahepatic lipid content; however, there was no significant alteration in the concentration of liver enzymes.^139^ Moreover, the KD exerts a more beneficial effect on NAFLD parameters than interventions such as calorie-restricted and low-fat diets. A small clinical trial revealed a greater reduction in intrahepatic TGs in patients with NAFLD after a 2-week carbohydrate-restricted diet without calorie restriction than in patients who received a calorie-restricted diet, without any significant weight-loss differences in the two treatment groups.^140^ Consistently, a 2-year multicenter trial that included more than 300 patients who enrolled in a comprehensive lifestyle modification regimen demonstrated similar weight-loss patterns with low-fat diets and LCDs, although the LCD group displayed superior HDL-C profiles.^141^

The KD might protect against NAFLD through several mechanisms^142^ (Fig. 3). On one hand, due to its low-carbohydrate content, the KD could decrease insulin levels, with a consequent increase in fat oxidation and reduced lipogenesis,^143^ and induce a microbiome shift, with increased folate production and limited inflammatory and oxidative stress.^54^ On the other hand, the KD-induced KBs may result in (1) satiety, which limits food intake and facilitates weight loss;^144^ and (2) epigenetic modifications, which play an important role in NAFLD pathogenesis. For example, β-HB increases the histone acetylation of genes that encode resistance factors against oxidative stress;^145^ (3) activates GPR109A, which is widely expressed in various types of immune cells and exerts anti-inflammatory effects in many diseases, including obesity, inflammatory bowel disease, and cancer;^109,146^ (4) inhibits NLRP3,^147^ a key inflammasome that activates pro-inflammatory cytokines, such as IL-1β and IL-18, which closely correlate with obesity and T2DM pathogenesis.^148^

### Polycystic ovarian syndrome

The polycystic ovarian syndrome (PCOS) is closely related to other metabolic and endocrinological abnormalities, including insulin resistance, hyperinsulinemia, T2DM, dyslipidemia, and hyperandrogenism, and is characterized by insulin resistance, androgen excess, and abnormal gonadotropin dynamics. Therefore, the treatment goal is to improve insulin resistance and weight loss and decrease luteinizing hormone (LH)/follicle-stimulating hormone (FSH) ratios and excess androgen levels (Fig. 3).

The KD has been postulated to have a positive impact on women with PCOS. A study implemented a 6-month period of the KD for women diagnosed with PCOS, with BMI greater than 27 kg/m^2^, and no other serious medical conditions. After 24 weeks, women with PCOS showed a significant decrease in fasting serum insulin (pre, post-design: 23.5 to post-design: 8.2), in the LH/FSH ratio (2.23 to 1.21), and the free testosterone level (2.19 to 1.70). Furthermore, participants had an overall mean weight loss of 12.1% and a mean 4.0 kg/m^2^ decrease in BMI.^149^ A crossover study compared the effects of a standard diet and an LCD on PCOS and showed that the LCD decreased glycemia, fasting serum insulin, and testosterone, and increased insulin sensitivity.^150^ Paoli et al. reported similar results, with significant reductions in BMI, glycemia, insulin, LDL-C, HDL-C, TGs, LH, testosterone, and dehydroepiandrosterone sulfate. The initial reversal of the LH/FSH ratio did not persist after 12 weeks.^151^ Although these studies demonstrated a beneficial effect of the KD on PCOS, they have limitations such as small sample size, broad age range, single-arm design, and a short intervention time interval.

The exact mechanism by which the KD achieves therapeutic benefits in PCOS remains unclear. The exact mechanism by which KD achieves therapeutic benefit on PCOS remains unclear. A variety of studies identify a central role of insulin resistance in the pathogenesis of PCOS. Fasting insulin shows a positive correlation with androgen levels in women with PCOS.^152^ Insulin stimulates increased production of androgens in theca cells isolated from women with PCOS, which effect is mediated by the insulin receptor.^153^ Furthermore, excess insulin inhibits liver sex hormone-binding globulin synthesis, which increased the delivery of free androgens to target tissue. It has also been reported that AMPK, a regulator of cellular metabolism and energy balance, plays an essential role in the improvements of KD toward PCOS.^154^

## Function in neurodegenerative diseases

### Alzheimer’s disease

Alzheimer’s disease (AD) affects ~50 million people worldwide and is characterized by cognitive impairment that is associated with a progressive decline in memory, impaired self-care, disorientation, and personality changes.^14^ AD induces changes in amyloid precursor protein cleavage and production of the amyloid precursor protein fragment beta-amyloid (Aβ) along with hyperphosphorylated tau protein aggregation.^155^ Patients with AD have mitochondrial dysfunction and metabolic changes, such as impaired glucose utilization in the brain.^156^ During the past decades, changes in dietary patterns and lifestyle modifications have had potential application in the treatment of AD and have received extensive attention in AD research, including calorie restriction, dietary approaches to stop hypertension, Mediterranean diet, and the KD.^157^

The KD is a biochemical model of fasting or starvation, which promotes the utilization of KBs as the dominant fuel source to replace glucose in the central nervous system.^158^ This may modulate the neuropathological and biochemical changes observed in AD, and the KD can directly reduce the accumulation of amyloid plaques while reversing Aβ toxicity; furthermore, KBs may protect against Aβ neurotoxicity.^159^ Kashiwaya et al. treated cultured rat hippocampal cells with Aβ, β-HB, or both Aβ plus β-HB. Treatment with Aβ alone resulted in reduced neurite numbers and length compared to controls, and the additional treatment with β-HB reversed Aβ toxicity, suggesting that β-HB could potentially play a neuroprotective role against Aβ toxicity.^160^

**Fig. 4:**
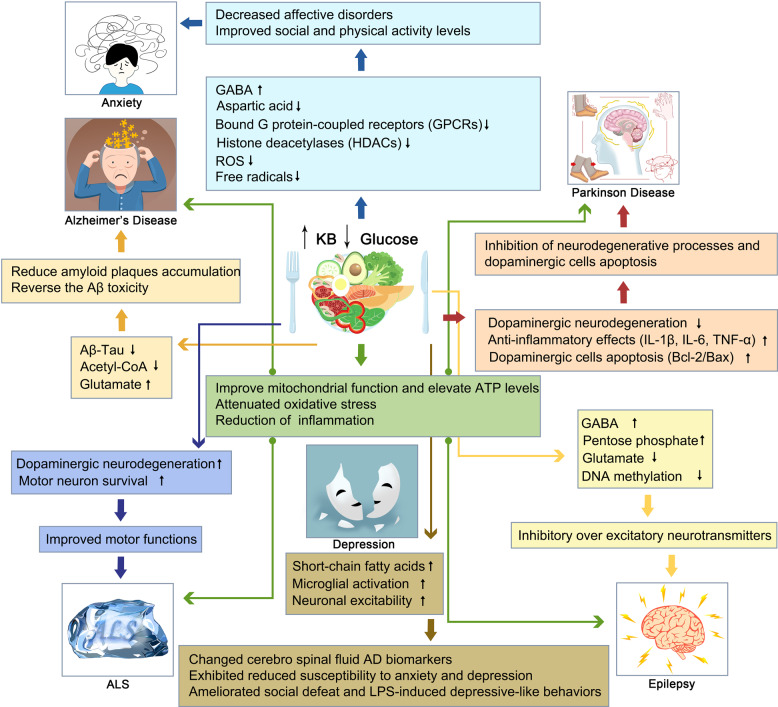
Mechanisms of the KD on neuromuscular and neurodegenerative diseases, including AD, PD, ALS, and epilepsy. Ketogenic diets altered the neuropathological and biochemical behavior through a variety of mechanisms including increasing mitochondrial function and ATP producing, decreasing oxidation stress and inflammation in the brain, and improving motor function and motor neuron survival

In addition, the development of AD is associated with hypometabolism, mitochondrial dysfunction, oxidative stress, and inflammation (Fig. 4).^161–164^ Impaired glucose uptake and utilization in regional brain energy-substrate hypometabolism may be one of the earliest hallmarks of AD and suggests a potential avenue for compensating the brain energy deficit in AD dementia with ketones.^165^ Both β-HB and acetoacetate bypass glycolysis to reduce acetyl-CoA, which can then be channeled into the Krebs cycle, and can thus increase energy availability in the brain.^166^ In AD, brain ketone uptake is unimpaired, which makes KBs a viable alternative energy source.

Mitochondrial dysfunction and oxidative stress play significant roles in neurodegenerative diseases, and both generate high levels of reactive oxygen species (ROS), which are harmful to all cellular macromolecules.^167^ Importantly, the KD could improve mitochondrial numbers and function by inhibiting glycolysis and increasing KB formation to provide neuroprotective benefits in the neuronal cell line (SH-SY5Y).^168^ In addition, KBs may regulate the homeostatic status of mitochondria by modulating calcium-induced membrane permeability transition.^169^ Moreover, the KD elevates brain ATP and induces a higher phosphocreatine/creatine ratio and glutamate levels, but decreases glycogen levels.^170^ Lu et al. reported that the KD increased superoxide dismutase activity and attenuated oxidative stress by activating Nrf2.^171^

Furthermore, the KD reduces inflammation through established effects. The β-HB receptor HCA2 could activate a neuroprotective phenotype of macrophages depending on PGD2 production by COX1 and hematopoietic PGD2 synthase.^172^ Pretreatment with the KD is associated with a reduction of pro-inflammatory cytokines, such as IL-1β and tumor necrosis factor alpha (TNF-α), which are induced by lipopolysaccharide injection, thereby suggesting that KD has anti-inflammatory properties.^173^ Another mechanism of KD is the inhibition of histone deacetylases (HDACs) by the β-HB.^163^ HDACs are a kind of protease that alters chromatin structure, and accessibility. HDACs are inhibited by β-HB, which improves memory function and synaptic plasticity.^170,174^ Moreover, the KD inhibits the activation of NF-κB in activated B cells and downregulates COX2.^77^ Thus, the KD could regulate dysregulated brain metabolism, mitochondrial homeostasis, and inflammation in AD.

In a transgenic mouse model of AD, the KD reduced the level of soluble Aβ deposits in the brain by 25% after only 40 days.^175^ In addition, treatment with the KD, exogenous β-HB, and MCT reduced brain Aβ levels and improved cognitive ability.^176^ When a mouse model of AD was fed a KD for 43 days, serum levels of the KB β-HB increased, total Aβ levels decreased, mitochondrial function improved, and oxidative stress decreased compared to controls.^177^ Aged dogs receiving MCT showed dramatically improved mitochondrial function and decreased oxidative damage compared to age-matched controls.^178^ In aged rats, the KD improved cognitive performance due to increased angiogenesis and capillary density, supporting the theory that diet-induced ketosis is beneficial in the treatment of neurodegenerative conditions.^179^

Clinical data show that significant increases in β-HB levels and improved memory were observed in 20 participants with AD or mild cognitive impairment when they were administered MCT.^180^ A randomized, double-blind, placebo-controlled, multicenter trial compared the effects of MCT on memory and cognition and demonstrated that elevated serum β-HB levels improved cognitive function and memory. Phillips et al. conducted a randomized crossover trial of a modified ketogenic diet in the treatment of Alzheimer’s disease. The results showed that patients achieved sustained physiological ketosis (12-week mean beta-hydroxybutyrate level: 0.95 ± 0.34 mmol/L). Compared with the usual diet, patients on the ketogenic diet increased their mean within-individual ADCS-ADL (*P* = 0.0067) and QOL-AD (*P* = 0.023) scores.^181^ Another study compared an LCD with a high-carbohydrate diet in 23 adult patients treated with MCTs for 6 weeks. The LCD showed better memory performance and was positively correlated with levels of KBs.^182^ Krikorian et al. reported maximum cognitive benefit of KD treatment in ApoE4(−) patients after 24-week treatment with MCT or a ketogenic product compared to placebo.^183^ This observational study was limited to ApoE4(−) patients with mild AD. In a recent study, MCT was administered to 20 Japanese patients with mild to moderate AD over 12 weeks, and 120 min after MCT intake, the level of KBs increased; after 8 weeks, the patients demonstrated significant improvement in their immediate and delayed memory tests compared to their baseline scores. Additional research is warranted to determine the therapeutic benefits of MCT in patients with AD and to ascertain how APOE-4 status may mediate β-HB efficacy.

### Parkinson’s disease

Parkinson’s disease (PD) is the commonest serious movement disorder worldwide and affects ~1% of adults who are older than 60 years.^184^ PD is characterized by the loss of nigrostriatal dopaminergic neurons and a deficit in mitochondrial respiration. α-Synuclein is an overexpression protein in PD while its relationship with PD is unknown. The knockout of α-Synuclein only increased the number of dopamine neurons which suggested that the loss of synucleins does not produce parkinsonism.^185^ In recent years, animal and in vitro studies have demonstrated the beneficial effect of KBs on the course of PD due to their function in mitochondrial homeostasis.

Joniec-Maciejak et al. observed that the administration of octanoic acid induced the inhibition of the neurodegenerative processes seen after 1-methyl-4-phenyl-1,2,3,6-tetrahydropyridine (MPTP) administration and was related to increased metabolic activity in striatal mitochondria.^186^ One of the important factors that cause PD is DNA oxidative damage mediated by reactive oxygen species. It has been discussed that the injury of the respiratory chain and the mutations of mitochondrial DNA found in patients with PD suggest the importance of oxidative stress in PD.^187^ The accumulation of hydroxyl radicals in the brain may lead to increased dopamine metabolism and, at the same time, to the accumulation of iron in the redox form of neurons.^188^ KBs produced by liver metabolism not only promote mitochondrial respiration by increasing ATP production, but also reduce free radical production by increasing the efficiency of the mitochondrial respiratory chain complex (increasing NADH oxidation and inhibiting mitochondrial permeability conversion). An increase in anti-peroxidase in the hippocampus protects the central nervous system from degenerative changes.^13,189^ Other studies showed that the KD exerted anti-inflammatory effects by decreasing the levels of pro-inflammatory cytokines, including IL-1β, IL-6, and TNF-α, in the substantial nigra.^190^ Cheng et al. found that β-HB could inhibit the apoptosis of dopaminergic cells that were exposed to MPTP in relation to the upregulation of Bcl-2/Bax mRNA.^191^ A summary of the KD-induced changes in PD is shown in Fig. 4.

In a rat model of PD, the KD, via glutathione activity, protected dopaminergic neurons of the substantia nigra against 6-hydroxydopamine neurotoxicity.^192^ In mice, β-HB infusion confers partial protection against MPTP-induced dopaminergic neurodegeneration and motor deficits.^193^ These effects appear to be mediated by a Complex II-dependent mechanism that improves mitochondrial respiration and ATP production. Shaafi et al. observed a beneficial influence of the KD on motor function in a rat model of PD and confirmed that, when coadministered, the KD potentiated the efficacy of pramipexole.^194^

In a clinical study, five patients with PD who consented to adhere to KD rules in their home environments were observed for 28 days and exhibited some improvements in the Unified Parkinson’s Disease Rating Scale.^195^ In another pilot RCT, the effect of a low-fat diet versus the KD in 47 PD patients was assessed; 44 participants commenced the diets, and 38 completed the study (86% completion rate). The KD group maintained physiological ketosis. Both groups showed significantly decreased MDS-Unified Parkinson’s Disease Rating Scale scores, but the KD group showed a greater decrease in Part 1 (−4.58 ± 2.17 points; 41% improvement in baseline Part 1 scores) compared to the low-fat group (−0.99 ± 3.63 points; 11% improvement) (*P* < 0.001); the largest intergroup differences in decreases were observed for urinary problems, pain and other sensations, fatigue, daytime sleepiness, and cognitive impairment. The results of the study indicated that it is plausible and safe for PD patients to maintain a low-fat diet or KD for 8 weeks. Both diet groups showed significant improvements in motor and nonmotor symptoms; however, the ketogenic group showed greater improvements in nonmotor symptoms.^196^

### Amyotrophic lateral sclerosis

Amyotrophic lateral sclerosis (ALS) is a neurodegenerative disease caused by the death of motor neurons in the spinal cord and brainstem, resulting in death within 2–5 years of symptom onset and respiratory paralysis. The accepted hypothesis of ALS pathogenesis includes loss of oxidative control with an excessive generation of oxidative free radicals, accumulation of neurofilaments, and excitotoxicity linked to an increase in the neurotransmitter glutamate, with resultant mitochondrial membrane dysfunction.^197^ Furthermore, at least 20 genes are associated with ALS. Most familial and sporadic cases of ALS are caused by variants of the superoxide dismutase 1 (SOD1), C9orf72, FUS, and TARDBP genes.^198^ To date, there are no effective treatments for ALS. The only US FDA-approved pharmacological therapy is riluzole, which could prolong survival by ~2 months in ALS patients.^199^

Mitochondrial dysfunction may play an important role in the pathogenesis of ALS, which makes it a potential novel therapeutic target of intervention for ALS. Kong showed that KD can increase antioxidant power and attenuate oxidative stress in spinal cord injury by suppressing class I histone deacetylases.^200^ Mutations in the gene encoding Cu/Zn SOD1 lead to abnormal mitochondrial activity, which is associated with a portion of ALS.^201^ In energy alterations secondary to the mitochondrial dysfunction in ALS, the KD increased energy production and regulated mitochondrial activity by restoring the activity of complex I of the electron chain whose function is reduced in ALS (Fig. 4).^202^

Moreover, the KD improved motor functions, which were associated with increased motor neurons in a mouse model of ALS. In the SOD1-G93A transgenic ALS mouse model, the KD led to higher motor neuron survival and an improvement in motor function compared to KO mice without the KD; furthermore, weight and synthesis of ATP at the mitochondrial level increased.^202^ Mice that were fed the KD exhibited better motor performance on all motor function tests at 15 and 16 weeks of age compared to controls and, after the implementation of the Deanna protocol based on the KD, significantly extended the survival time of SOD1-G93A mice; 63% of mice in this group lived beyond 125 days, whereas only 9% of the control animals survived beyond this timepoint.^203^ In another study, SOD1-G93A mice were treated with caprylic triglyceride, a medium-chain TG that is metabolized into KBs and can serve as an alternative energy substrate for neuronal metabolism. Treatment with caprylic triglyceride attenuated the progression of weakness, protected spinal cord motor neuron loss, and promoted the mitochondrial oxygen consumption rate in vivo.^204^ These studies indicate that KD treatment may have a high impact on the quality of life of ALS patients.

### Epilepsy

Epilepsy is a chronic brain disorder characterized by recurrent seizures, which are short episodes of involuntary movement that can affect a part or all of the body, and are sometimes accompanied by loss of consciousness or loss of control of bladder or bowel function. The causes of epilepsy include brain tumors, stroke, brain infection, severe head injury, congenital abnormalities associated with brain defects, brain damage due to prenatal or perinatal injuries, and certain genetic syndromes.^205^ Although 50–70 million people worldwide suffer from epilepsy, the available pharmacological treatment for epilepsy has limited effectiveness.^206^

The KD was first recognized as an effective treatment for epilepsy. Studies have re-emerged that demonstrating the efficacy of KD in patients with drug-resistant epilepsy and some pediatric epilepsy syndromes.^207^ The anticonvulsant mechanisms of the KD are incompletely understood, and the potential mechanisms of the KD are based essentially on the role of neurotransmitters, brain energy metabolism, ion channels, and oxidative stress (Fig. 4).^7^ One study confirmed that norepinephrine and the orexigenic neuropeptide galanin are the two classes of molecules that contribute to the anticonvulsant effect of the KD.^208^ However, in children with refractory epilepsy, the KD does not alter the level of norepinephrine but significantly alters the levels of dopamine and serotonin metabolites in the cerebrospinal fluid.^209^ And a significant increase in γ-aminobutyric acid (GABA) and agmatine levels, without changes in glutamate levels, was observed in the hippocampus of rats subjected to a KD for 15 days compared to rats on a normal rat chow diet; this supports the notion that the KD modifies different transmitters while favoring inhibitory over excitatory neurotransmitters.^210^ Recently, Zarnowski et al. find that the KD plays an important role in the neuroprotection and anti-convulsion via the kynurenine pathway. Kynurenic acid, the metabolite from the kynurenine pathway, participates in epilepsy. KBs, rich in KD, can inhibit glutamate which reduces the production of kynurenic acid.^211^ While ATP is produced by mitochondrial respiration, the damaged substance ROS is produced at the same time. The uncoupling proteins, upregulated by mitochondrial respiration, can reduce the production of ROS and increase the resistance towards seizure. Moreover, the KD can also protect the mitochondrial DNA from ROS by improving the glutathione levels. In final, the KD can generate beneficial substances to reduce the happening of epilepsy.^212,213^ Under KD conditions, the reduction of brain glucose utilization and glycolytic ATP production may induce potassium channels that are sensitive to ATP opening, which leads to the hyperpolarization of the neuronal membrane,^214^ consequently reducing the electrical excitability of the brain and increasing the seizure threshold. Moreover, KD treatment increases polyunsaturated fatty acids and induces the expression of neuronal uncoupling proteins, regulates numerous energy metabolism genes, and induces mitochondrial biogenesis, which further limits ROS generation and increases energy production.^215^

A meta-analysis reviewed 12 studies using classic KD, MAD, and classic KD in combination with MCT in adults with antiepileptic drug-resistant epilepsy. They found a combined efficacy rate of 52% for classic KD and 34% for MAD. The results indicate that a classical ketogenic diet may be more effective, and adult patients are likely to be less compliant with the KD than with an MAD.^216^ In an observational study, at 3 months, 36% of 101 participants responded (≥50% seizure reduction) to diet therapy, and 16% were seizure-free. At 1 year, 30% responded, and 13% were seizure-free. At 4 years, 21% responded, and 7% were seizure-free. This study provided evidence that ketogenic diets may be feasible, effective, and safe in the long-term in adults, although long-term adherence was limited. Thus, adequately controlled studies are necessary to determine the efficacy of ketogenic diets in the treatment of adults with epilepsy.^217^ An RCT in Iran compared the proportion of patients with focal or generalized epilepsy achieving ≥50% seizure reduction between 34 patients randomized to 2 months of MAD (22 completed the study), compared to 32 patients randomized to standard medical management, and found 35.5% (12/34) efficacy in the MAD group (intention-to-treat (ITT) analysis) at 2 months compared to 0% in the control group, a difference that was statistically significant.^218^ In contrast, an RCT in Norway compared the change in seizure frequency in patients with drug-resistant focal or multifocal epilepsy between 37 patients randomized to 12 weeks of MAD (of whom 28 received the intervention and 24 completed the study) and 38 adults randomized to their habitual diet (of whom 34 received the intervention and 32 completed the study).^219^ Overall, ITT data from adult observational studies demonstrate responder rates of 22–70% for classic KD and 12–67% for MAD, with some suggestion of increased efficacy in adults with generalized rather than focal epilepsy.^220,221^ Additional RCTs with larger sample sizes are warranted to investigate the efficacy of the MAD in different subpopulations of adult patients with epilepsy. Thus, the KD offers a necessary adjunctive strategy for management with the advantages of potentially synergizing concurrent treatments and reducing the need for prolonged use of anesthetic drugs.

### Depression

Depression is a common kind of mood disorder that involves reduced inhibitory GABA neurotransmission. KD may do a favor to depression via its nutrition and microbiome that attracts more attention from doctors and researchers. The main source of GABA is the glutamate–glutamine cycle which happens to be adequate in KD. Moreover, KD is full of other nutrition such as ω-3s, tryptophan, vitamin B, which may regulate the disorders in both physiology and psychology.^222^ The gut–brain axis suggests that the microbiome plays an important role in mental health and an existing study proves that the KD may affect mental health through the gut–brain axis by the gut microbiome. Nagpal et al. find that the KD alters the gut microbiome signature and short-chain fatty acid in association with cerebrospinal fluid AD biomarkers.^223^

KD has been reported to exhibit preventative effects on depressive-like behaviors in rodents while its exact mechanism is still unknown. As we noticed before, the adult offspring of pregnant mice fed by KD exhibited less prone to depression behaviors.^224^ Recently, Sun et al. find that a ketogenic diet can ameliorate social defeat and lipopolysaccharide-induced depressive-like behaviors by the restoration of microglial inflammatory activation and neuronal excitability.^225^ Also, Campbell’s report revealed that a ketogenic diet may lead to mood stabilization.^226^ According to the randomized controlled trial, DM. et al. find that the KD group showed lower levels of anxious and mood-disturbed behaviors.^227^

### Anxiety disorders

Anxiety disorder is an increasingly common mental disorder worldwide, resulting in considerable mental stress for patients, and social medicine and economic burden. According to epidemiological statistical analysis, about one-third of the global population is affected by anxiety disorders during their lifetime.^228^ Current treatments including drugs, psychological interventions, and so on still fail to meet the need for a cure. Recently, nutritional therapies such as ketogenic diets are considered promising due to their effectiveness in preventing relapse.

Disorders in the metabolism of neurotransmitters are considered to be a major factor in anxiety disorders. A deficiency of the central inhibitory neurotransmitter GABA has been linked to anxiety, depression and other affective disorders. Due to the decrease of blood glucose level during the implementation of the ketogenic diet, the glycolysis pathway in the brain is significantly inhibited, and the energy supplier of the central nervous system changes from glucose to ketone bodies. Excitation of the steroid pathway enhances the synthesis and transmission of GABA at the synapse, while decreasing the content of aspartic acid and the excitability of neurons.^229,230^ It is suggested in several studies that the progeny of mice exposed to KD during pregnancy show lower susceptibility to anxiety and depression and significantly improve social and physical activity levels compared to the standard diet group.

On the other hand, bi-directional interactions between the central nervous system and the intestinal microbiome are associated with the development of psychiatric disorders. With the disorder of intestinal microflora, the barrier function of the intestinal epithelium is gradually reduced or even lost with the increased permeability, which then mediates the enhancement of immune response and causes chronic neuritis. Neurotransmitter metabolism, particularly serotonin metabolism, which is closely associated with the onset of anxiety and depression, would be disrupted.^231^ Mechanistically, ketones produced by liver metabolism act as both energy supply molecules and signal molecules involved in binding G protein-coupled receptors, inhibiting HDACs, regulating the abundance of intestinal microbiota, improving intestinal barrier function, and reducing the production of ROS and free radicals.^232,233^ Experiments on mice have demonstrated the positive effects of KD on the intestinal microbiome.^234^ Overall, KD has an ideal potential to be further used to the mood disorder and the mechanism behind remains to be explored.

## Function in cancer

Cancer is one of the greatest global public health challenges and is a leading cause of global mortality. Complementary approaches to significantly enhance the efficacy of standard anticancer therapies are scarce. The KD appears to sensitize most cancers to standard treatment by exploiting the reprogramming metabolism of cancer cells, making it a promising candidate in adjuvant cancer therapy.^17^

Tumor cells use glucose as the primary energy source. To meet the requirements of rapid proliferation, tumor cells utilize glycolysis, even in the presence of oxygen: a phenomenon known as the “Warburg effect”.^235^ Thus, any pharmacologic intervention that reduces intratumoral glucose levels may be effective for slowing tumor growth. During KD implementation, tumor cells have limited access to glucose and cannot use KBs as an energy source owing to aberrant mitochondrial function and reduced enzymatic activity for ketone consumption, which makes the KD a promising approach for cancer prevention. Due to a reduction in blood glucose levels, the KD could concurrently affect glucose metabolism and glucose-dependent signaling in tumor cells.^236^ Furthermore, glucose starvation leads to a suppressed lactate/pyruvate cycle, which inhibits neovascularization, hypoxia-induced vascular epidermal growth factor activation, and angiogenesis, and causes ultimate necrosis in tumor cells, especially for colon adenocarcinoma xenografts.^237^ KBs can inhibit inflammation, which is closely correlated with tumor pathogenesis. By inhibiting the NLRP3 inflammasome, β-HB diminishes the inflammatory microenvironment, which provides ancillary therapeutic benefits for therapeutic interventions in glioblastoma.^238^ Interestingly, GPR109A, a receptor for β-HB, is downregulated in cancer. GPR109A, a tumor suppressor, was downregulated when β-HB synthesis was suppressed. Therefore, low levels of β-HB attenuate the tumor-suppressive function of GPR109A in colon cancer cells.^239^ Recent studies have extended the tumor-suppressive function of the receptor beyond the colon, as GPR109A suppressed mammary tumorigenesis in a mouse model of breast cancer.^240^

Furthermore, the KD could enhance the efficacy of phosphatidylinositol 3 kinase (PI3K) inhibitors and overcome drug resistance, which was confirmed in different mouse cancer models, including pancreatic, bladder, endometrial, and breast cancer, as well as in acute myeloid leukemia. The KD improves the efficacy of anti-PI3K treatment and drug resistance by decreasing hyperglycemia and reducing the insulin-secretory response, and these effects correlated with reduced intratumoral mTORC1 signaling.^241^ In a mouse model of melanoma, acceleration of proliferation in BRAF V600E-mutated melanoma cells after the KD treatment was observed because of the selectively increased activation of BRAF V600E-mutant-dependent MEK1 signaling by the KB acetoacetate. In contrast, NRAS Q61K- and Q61R-mutated and BRAF wild-type melanoma cells were unaffected by the KD.^242^

Besides slowing tumor growth, KD sensitizes tumor cells to classic chemotherapy or radiotherapy in neuroblastoma, glioma, and lung cancer.^243–246^ For example, the KD supplemented with medium-chain TG enhanced the antitumor and anti-angiogenic efficacy of chemotherapy on neuroblastoma xenografts in a CD1-nu mouse model.^247^ In addition, KD showed promising benefits in boosting the effect of anti-PD-1/PD-L1 immunotherapy. Ferrere et al. found that anti-PD-1, alone or in combination with anti-CTLA-4, failed to inhibit tumor growth in mice receiving a standard diet, whereas KD implementation, or oral administration of 3-hydroxybutyrate (3-HB), a principal KB generated in the KD, reestablished therapeutic responses.^248^ 3-HB prevented the upregulation of PD-L1 on myeloid cells whereas promoting the expansion of CXCR3^+^ T cells and instituting consequent T cell-mediated tumor immunosurveillance. Similarly, Dai et al. reported that KD treatment decreased PD-L1 protein levels and increased the expression of type-I interferon (IFN) and antigen-presentation genes, leading to the enhanced efficacy of anti-CTLA-4 immunotherapy.^249^ The key molecular event of KD treatment in promoting immunotherapy is the activation of AMPK, which phosphorylates PD-L1, resulting in its disrupted interaction with CMTM4 and subsequent PD-L1 degradation, which phosphorylates EZH2, resulting in improper PRC2 function and subsequently enhanced expression of IFN and antigen-presentation gene.^249^

Many studies investigating the effect of the KD on tumor metastasis indicate a metastasis-inhibiting effect of the KD.^250–252^ An early study reported that KBs could inhibit the growth rate of malignant lymphoblasts (Raji cells) and diet-induced ketosis could reduce the number of B16 melanoma deposits in mouse lung.^251^ Combined treatment with the KD and hyperbaric oxygen significantly reduced the tumor-growth rate and diminished metastatic spread, while increasing survival, in the VM-M3 mouse model of metastatic cancer, possibly through induction of ROS production in tumor cells.^250^

Apart from preclinical studies, clinical trials have demonstrated the beneficial effects of the KD on antitumor therapy. A clinical trial showed that positron emission tomography revealed an average decrease of 21.8% in glucose uptake at the tumor site in children after 8 weeks of KD implementation. One child displayed significant mood improvement, promotion of skill-learning ability, and remained free of disease progression after continuing the KD for 12 months. All participants remained in remission for 4–5 years after diagnosis, with good quality of life. A prospective feasibility trial applying the MAD to patients with glioblastoma who were not receiving chemotherapy reported that four patients were stable or improved after 16 weeks of dietary intervention.^253^ A randomized controlled clinical trial showed that TNF-α decreased significantly after 12 weeks of treatment (*P* < 0.001), while IL-10 increased (*P* < 0.001) in the intervention compared to the control group. Patients in the KD group had lower adjusted serum insulin compared to the control group (*P* < 0.002). KD lead to a reduction in tumor size in the KD compared to the control (27 vs 6 mm, *P* = 0.01). Stage decreased significantly in patients with locally advanced disease in the KD group after 12 weeks (*P* < 0.01).^254^ A study that investigated the effect of KD in combination with intranasal perillyl alcohol in patients with recurrent glioblastoma found that after 3 months of combined therapy, the KD increased the partial response rate to 77.8%, compared with 25% in the control group. The percentage of tumor progression was 11.1% in the KD group, compared to 50% in the control group.^255^ A study among ovarian and endometrial cancer patients reported that the KD improved overall physical health and increased energy in patients without chemotherapy.^256^ Therefore, the administration of KD may be a potential approach to enhance the therapeutic efficacy of chemotherapy. In addition, the boundary between a high-fat diet and KD still needs to be further clarified. Recently, Jun Yu’s team found that high-fat diet can drive colorectal tumors by inducing gut microbiota dysbiosis, metabolic changes, and intestinal epithelial barrier dysfunction in mice, and revealed potentially key bacteria and metabolites. Moreover, compared with a high-oleic acid diet, a high-fat diet rich in linoleic acid can specifically promote breast tumor growth.^257^

**Fig. 5:**
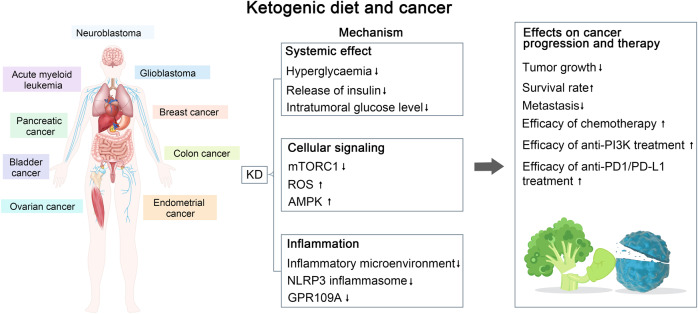
Summary of the potential interplay in the molecular mechanisms of the ketogenic diet (KD) and cancer. The KD exerts a therapeutic effect on tumors such as neuroblastoma, acute myeloid leukemia, glioblastoma, etc., through decreased GPR109A expression, mTORC1 activation, and glucose uptake at the tumor site, which leads to decreased tumor growth, increased survival, and increased chemotherapeutic efficacy

Taken together, the KD has shown benefits in tumor-growth inhibition and enhanced efficacy of multiple antitumor therapies in various types of cancer, including glioblastoma, prostate, colon, pancreatic, and lung cancer (Fig. 5). These mechanisms can be attributed to a limited glucose source and reduced inflammation. However, the efficacy of KD could be influenced by cancer type, subtype, genetic features, or tumor-associated syndrome. Future studies should focus on more molecular studies as well as RCTs with heterogeneous study designs and large samples in order to elucidate the mechanisms of the KD in tumor therapy and to evaluate the application of the KD in the clinical setting.

## Function in cardiac diseases

Heart failure (HF) is characterized by metabolic abnormalities. Therefore, augmenting cardiac ATP production in a bioenergetically efficient manner is of significant interest to the HF field.

Growing evidence has demonstrated increased ketone body utilization in the failing heart. For example, expression of β-hydroxybutyrate dehydrogenase 1 (a key enzyme in the ketone oxidation pathway) and ketogenic β-hydroxybutyryl-CoA, in association with increased myocardial utilization of β-hydroxybutyrate, is increased in the heart failure samples supported the notion that the hypertrophied and failing heart shifts to ketone bodies as an alternate fuel and myocardial ketone oxidation as a key metabolic adaptation in the failing human heart.^258,259^ In mice and canine pacing model of progressive heart failure, increased delivery of 3-HB ameliorates pathologic cardiac remodeling and dysfunction. Mice rendered incapable of oxidizing the ketone body 3-HB in the heart exhibits worsened heart failure compared with WT controls. These results indicate that the heart utilizes 3-HB as a metabolic stress defense and suggest that strategies aimed at increasing ketone delivery to the heart could prove useful in the treatment of heart failure.^260^ Preclinical and clinical studies also suggest that exogenous delivery of ketones may improve cardiovascular function as well as prevent the development of pathological remodeling en-route to HF.^261^ The potential mechanism of ketosis is associated with SGLT2 inhibitors and reductions in HF morbidity and cardiovascular mortality were observed in patients with HF (irrespective of diabetes status) further confirmed the potential benefits of ketones in patients with HF.^262^

Zhang et al. have constructed mice with cardiomyocyte-restricted deletion of subunit 1 of MPC (cMPC1^−/−^). The mice develop age-dependent pathologic cardiac hypertrophy, transitioning to a dilated cardiomyopathy and premature death. The KD could increase the availability of non-glucose substrates in vivo and reverse the structural, metabolic, and functional remodeling of non-stressed cMPC1^−/−^ hearts. Although concurrent short-term KD did not rescue cMPC1^−/−^ hearts from rapid decompensation and early mortality after pressure overload, 3 weeks of the KD before transverse aortic constriction is sufficient to rescue this phenotype.^263^

Although the beneficial effects of β-HB on HF have been acknowledged by numerous reports, its safety on other cardiovascular problems has been challenged by certain lines of evidence. In rat models, KD decreased mitochondrial biogenesis, reduced cell respiration, and increased cardiomyocyte apoptosis and cardiac fibrosis. Mechanistically, the KD increases levels of β-HB, promotes histone acetylation of the Sirt7 promoter, and activates Sirt7 transcription. This in turn inhibits the transcription of mitochondrial ribosome-encoding genes and mitochondrial biogenesis, leading to cardiomyocyte apoptosis and cardiac fibrosis.^264^ This study highlighted the unknown detrimental effects of the KD.

In atrial fibrillation which is the most common arrhythmia encountered in clinical practice, the concentration of β-HB in heart tissues is significantly higher.^265^ The potential detrimental effects of β-HB have also been confirmed in clinical studies. A retrospective study assessed the relationship between serum β-HB and prognosis in 405 stable hemodialysis patients. Increased serum β-HB levels are independently associated with cardiovascular events and all-cause death. In addition, increased circulating β-HB is independently associated with major adverse cardiovascular events.^266^ Twenty patients on the ketogenic diet at one institution were investigated in another research. Prolonged QT interval was found in three patients (15%). There was a significant correlation between prolonged QT interval and both low serum bicarbonate and high beta-hydroxybutyrate. In addition, three patients had evidence of cardiac chamber enlargement. One patient with severe dilated cardiomyopathy and prolonged QT interval normalized when the diet was discontinued.^267^ Taken together, these findings suggest that KD consumption or β-HB accumulation may increase the risks of cardiovascular disease, suggesting that long-term consumption of a KD should be carefully considered in cardiovascular disease.

**Fig. 6:**
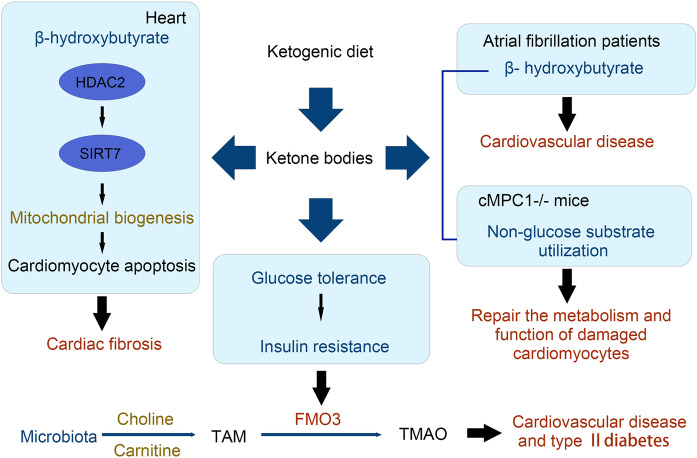
Improvements and mechanisms of the functions of the KD exert on cardiac diseases. The KD increases levels of β-HB, promotes histone acetylation of the Sirt7 promoter and activates Sirt7 transcription in cardiac fibrosis and increases the availability of non-glucose substrates in cMPC1^−/−^ hearts

By digesting animal protein and other components of animal products—red meat—symbiotic bacteria in the gut produce metabolites that have been linked to insulin resistance and cancer formation. One such molecule, trimethylamine N-oxide (TMAO), has recently gained a lot of attention as a possible and a closely linked risk factor for gut microbiota and cardiovascular and kidney disease. Trimethylamine is produced by gastrointestinal bacteria after they metabolize dietary choline and carnitine. Trimethylamine is then absorbed and oxidized to TMAO under the action of flavin-monooxygenases (FMOs), mainly FMO3.^268^ KD changes the pattern of energy metabolism and makes more use of fat and ketone bodies through a very-low-carbohydrate and high-fat diet. The researchers compared the ketogenic diet in mice with the obesity-inducing high-fat diet and found that the ketogenic diet, while apparently healthier, significantly reduced glucose tolerance. The liver of the mice became less responsive to insulin and became insulin resistant.^269^ In people with hepatic insulin resistance, low hepatic insulin activity increases FMO3 expression, further enhancing TMAO levels. Hepatic insulin resistance, often associated with hepatic steatosis, may lead to increased cardiovascular risk and an increased risk of type 2 diabete, and these increased risks are also associated with elevated TMAO (Fig. 6).

## Function in other inflammation diseases

### Inflammatory bowel disease

A recent study found that the KD can alleviate colitis in a dextran sulfate sodium mouse model compared with a low-carbohydrate diet and normal diet.^20^ A 16-week KD intervention before colitis induction in mice was found to increase the abundance of *Akkermansia* and *Roseburia* and simultaneously alter the gut metabolites. Upon DSS treatment, the KD-fed mice had decreased weight loss and disease activity index scores with reduced inflammatory cell infiltration in the intestinal epithelium. The KD was found to exert an anti-inflammation effect via reducing the production of RORγt^+^CD3^−^ group 3 innate lymphoid cells as well as the related inflammatory cytokines including IL-17α, IL-18, IL-22, and CCL-4. The KD-fed mice remained to have overabundant *Akkermansia* in feces. Importantly, transplantation of the feces of KD-fed mice to germ-free mice retained the above effects, highlighting the KD-modulated gut microbiota have a profound role in alleviating colitis.

### Irritable bowel syndrome

A study also showed that KD contributes to beneficial effects in a rat model of irritable bowel syndrome via reducing the stress on gut mitochondrial biogenesis.^270^ Feeding with KD was found to be able to reduce intestinal inflammation, improve cellular redox status and restore mitochondrial function in the model. Such effects might be exerted due to the upregulation of the PPAR-γ/PGC-1α axis (Table 2).

**Table 2:** Main improvements and underlying mechanisms of the KD exerts on the diseases below

Author, year	Diseases		Improvements	Underlying mechanisms
Gannon et al.^93^; Nuttall et al.^94^; Dashti et al.^95^; Hussain et al.^96^	T2DM		Reduction of blood glucose	Glucose transporter type 4 and O-GlcNAc-modified proteins may be involved
			Reduction of hemoglobin A1c	
			Reduction of blood insulin level	
			Improved insulin resistance	
Westerterp-Plantenga et al.^119^; Veldhorst et al.^120^; Sumithran et al.^121^; Johnstone et al.^122^; Laeger et al.^123^	Obesity		Increased satiety	Increased concentrations of “satiety” regulating hormones and direct suppression of appetite by ketone bodies
Yang et al.^124^			Reduction in lipogenesis	Improved insulin resistance
Ma et al.^125^			Increased lipolysis	Increased expression of lipolytic enzymes
Tagliabue et al.^126^; Paoli et al.^127^			Higher metabolic efficiency in consuming fats	Reduction in the resting respiratory quotient
Fine & Feinman^128^; Feinman & Fine^129^			Higher energy cost	Increased energy consumption in gluconeogenesis and the thermic effect of protein digestion
Paoli et al.^118^	NAFLD		Increased fat oxidation and reduced lipogenesis	Decreased insulin level
Shimazu et al.^145^			Increased oxidative stress resistance	β-HB increases histone acetylation of genes encoding oxidative stress resistance factors
Taggart et al.^109^; Graff et al.^146^; Youm et al.^147^			Reduction in hepatic inflammation	Activation of GPR109A and inhibition of NLRP3
Mardinoglu et al.^54^			Increased folate production	Microbial alteration of the gut microbiota
Mavropoulos et al.^149^; Gower et al.^150^; Paoli et al.^151^	PCOS		Reduction of LH/FSH ratio	Unclear; AMPK may be involved
			Reduction of testosterone level	
			Reduction of blood insulin level	
Broom et al.^159^; Kashiwaya et al.^160^	AD		Reduces amyloid plaques, and reverses Aβ toxicity	Increased neurite number and length
Hughes et al.^168^; Kim et al.^169^; Bough et al.^170^			Improved mitochondrial function and elevated ATP levels	Improves the number and function of mitochondria; modulates the calcium-induced membrane permeability transition (mPT)
Lu et al.^171^			Attenuated oxidative stress	Nrf2 activation
Cullingford et al.^77^; Rahman et al.^172^; Dupuis et al.^173^			Reduction of inflammation	Reduction of pro-inflammatory cytokines, such as IL-1β and TNF-α, inhibited the activation of NF-κB in activated B cells and downregulated COX2 expression
Joniec-Maciejak et al.^186^	PD		Inhibition of neurodegenerative processes increased metabolic activity in striatal mitochondria	
Yang & Cheng^190^			Anti-inflammatory effects	Decreased pro-inflammatory cytokine expression, including IL-1β, IL-6, and TNF-α, in the substantia nigra
Cheng et al.^191^			Inhibition of dopaminergic cell apoptosis	Upregulation of the Bcl-2/Bax ratio
Kong et al.^200^	ALS		Attenuation of oxidative stress	Suppression of Class I histone deacetylases
Zhao et al.^202^			Regulated mitochondrial dysfunction	Restores the activity of Complex II of the electron chain
			Improved motor functions	
Weinshenker et al.^208^; Dahlin et al.^209^; Calderón et al.^210^	Epilepsy		Prioritizes inhibitory over excitatory neurotransmitters	Increased norepinephrine and orexigenic neuropeptides, galanin metabolites of dopamine and serotonin, GABA, and agmatine
Yellen et al.^214^			Reduced brain glucose utilization and glycolytic ATP production	Induces potassium channels sensitive to ATP opening
Andrews et al.^215^			Limited the ROS generation	Increased polyunsaturated fatty acid levels and induced the expression of neuronal uncoupling proteins
Nagpal et al.^223^	Depression		Changed cerebrospinal fluid AD biomarkers	Modulated gut microbiome and short-chain fatty acids
Sussman et al.^224^			Exhibited reduced susceptibility to anxiety and depression	Programed the offspring neuro-anatomy and influences their behavior in adulthood
Campbell et al.^226^; DM et al.^227^			Ameliorated social defeat and lipopolysaccharide-induced depressive-like behaviors	Restoration of the microglial activation and the neuronal excitability in the lateral habenula
Forte et al.^229^; Erecińska et al.^230^ Sussman et al.^224^	Anxiety disorders		Decreased affective disorders, and improved social and physical activity levels	Enhances the synthesis and transmission of GABA at the synapse, decreases the content of aspartic acid and the excitability of neurons
Rawat et al.^231^; Cheng et al.^232^; Youm et al.^147^			Regulated the abundance of intestinal microbiota, and improved intestinal barrier function	Bound G protein-coupled receptors, inhibit histone deacetylases (HDACs) and reduced the production of ROS and free radicals
Hao et al.^237^; Vallejo et al.^285^	Cancer	Colon adenocarcinoma, glioblastoma	Affected glucose metabolism	Suppresses the lactate/pyruvate cycle, inhibits neovascularization and activates hypoxia-induced vascular epidermal growth factor and angiogenesis
Shang et al.^238^; Ristic et al.^239^; Elangovan et al.^240^		Glioblastoma, colon carcinoma, breast cancer	Inhibited inflammation	Inhibits NLRP3 inflammasome, GPR109A, which is a receptor for β-HB, which is downregulated in cancer
Hopkins et al.^241^; Xia et al.^242^		Pancreatic, bladder, endometrial, breast cancer, acute myeloid leukemia	Overcomes drug resistance	Decreased hyperglycemia and insulin secretion, reduced intratumoral mTORC1 signaling, selectively increased activation of *BRAF V600E*-mutant-dependent MEK1 signaling
Morscher et al.^243^; Allen et al.^244^; Abdelwahab et al.^245^; Zahra et al.^246^; Ferrere et al.^248^; Dai et al.^249^		Neuroblastoma, glioma, lung cancer	Improved the efficacy of classical chemotherapy or radiotherapy, anti-PD1/PD-L1 immunotherapy, and anti-CTLA-4 immunotherapy	Anti-angiogenic efficacy prevented the upregulation of PD-L1, promoted the expansion of CXCR3 + T cells and consequent T cell-mediated tumor immunosurveillance, decreased PD-L1 protein levels, and increased the expression of type-I interferon and antigen-presentation genes
Poff et al.^250^; Magee et al.^251^; Poff et al.^252^		Acute myeloid leukemia, melanoma	Inhibited tumor metastasis	Induction of ROS production in tumor cells

### Covid-19

The covid-19 pandemic is now a global threat. Especially, severe covid-19 with cytokine storm syndrome is desperately lethal and a main cause of mortality. The KD has been proposed as an adjunct therapy for covid-19 patients due to its contribution to the reduction of critical risk factors such as obesity, type 2 diabetes and hypertension, anti-inflammation, and metabolism modulation.^21,271^ It is proposed that switching lipid metabolism, which can be achieved by a ketogenic diet rich in MCTs or by intermittent fasting, may disfavor viral replication and infection and inhibit cytokine storm.^272^ Importantly, it is also believed that the induction of ketosis may help to prevent the cytokine storm.^273,274^ A recent retrospective analysis of 34 covid-19 patients receiving an eucaloric KD in comparison to 68 who received a eucaloric standard diet showed that the former might have a lower risk in motility and ICU admission.^275^ Thus, the KD could be a theoretical preventive and supportive care option for patients with covid-19. But clinical evidence are needed.

### Adverse effects and challenges

Short-term adverse effects, such as fatigue, irritability, headache, nausea, dehydration, hypoglycemia, diarrhea, metabolic acidosis, and refusal to eat, are commonly seen during the first few weeks of the KD as responses to diet-induced metabolic shift and are usually predictable and preventable.^276,277^ Long-term adverse effects of KD include elevated cardiovascular risks with poor cholesterol profiles and nephrolithiasis, likely due to the metabolic effects of the KD, such as uric acidemia, hypocitraturia, hypercalciuria, aciduria, growth retardation, decreased bone mineral density, anemia, and neuropathy,^276,277^ and are usually monitored during follow-up. Furthermore, there are adverse effects that can span the duration of KD therapy, which frequently include gastrointestinal disturbances, including constipation, abdominal pain, emesis, and gastroesophageal reflux disease.^277^ Due to the lack of sufficient clinical evidence of the long-term safety of the KD, the use of KD in chronic diseases remains debatable.^276^

Moreover, due to the lack of sufficient clinical evidence demonstrating the efficacy and safety of KD, there still are debates on using KD in several diseases,^276^ including diabetes, cardiovascular disease, and Alzheimer’s disease. As type 1 diabetes is often associated with metabolic irregularities, hyperketonemia and ketosis may increase the risk of complications.^278^ Risks of KD in certain diseases are also demonstrated in animal models. In spontaneously hypertensive rats, the KD increases hypertension,^279,280^ impairs endothelium-dependent relaxation in mesenteric arteries,^280^ causes renal damage^279^ and aggravates interstitial fibrosis and inflammatory responses in the heart.^281^ In the rat, The KD exacerbated disorders of glucose and lipid metabolisms and activated the renin-angiotensin-aldosterone system,^279^ and the ketone body might reduce eNOS expression via NF-κB singling pathway in vein endothelial cells,^280^ which could collectively contribute to hypertension and endothelial dysfunction. Meanwhile, KD reduced renal autophagy to cause damage and the β-hydroxybutyrate was found to promote TGF-β-induced fibrosis in cardiac fibroblasts.^281^ The KD can be detrimental to cognitive behavior. In an Alzheimer’s disease rat model, KD worsened cognitive performance likely because it exacerbated gut dysbiosis.^282^ Consistently, a very recent study showed that the KD and hypoxia-altered gut microbiota increased intestinal IFN-γ-producing Th1 cells and consequently impaired cognitive behavior in mice.^86^ Also, in an animal model of early Parkinson’s disease, long-term KD seemed to be insufficient for the neuroprotective effect.^283^

Furthermore, the KD is a multidisciplinary therapy that requires the involvement of experienced caregivers, such as doctors, nurses, and dietitians, in addition to the patients.^284^ Thus, successful implementation of the KD requires well-organized and consistently functioning cooperation between caregivers and patients, and clinical settings that support this cooperation. Moreover, as a patient-centered therapy, treatment adherence remains a major challenge of the KD.^277,284^ Ineffectiveness, adverse effects, diet restrictiveness, and unpalatable taste may decrease the patient’s motivation and lead to the discontinuation and consequent failure of the treatment.

### Future directions

Dietary interventions for the treatment and prevention of diseases are now widely acknowledged and have increasingly gained importance. Diet planning may not only serve as a medical approach for the treatment of diseases but can also provide a way to maintain health in the general population. The KD, as a potential therapeutic approach for various diseases, still faces challenges with regard to broad clinical application. Future studies are needed to provide high-quality clinical evidence on the efficacy and safety of the KD in diseases other than epilepsy; to further modify the diet to decrease the adverse effects and increase tolerability; and to comprehensively understand the mechanisms that may, in turn, guide disease- or patient-customized application and even a KD-derived drug design.

The KD exhibits broad therapeutic potential in many diseases other than epilepsy; however, the clinical implementation of the KD is not well established yet. High-quality RCTs must be conducted to confirm the safety and efficacy of the KD. In addition, based on clinical research, it is important to identify the syndromes and conditions that can benefit from the KD-based therapies, contraindications for use, KD variants with good effectiveness and tolerability, and factors and biomarkers that can predict KD-related outcomes, which may collectively guide the clinical utilization of the KD in different diseases.

Despite the adverse effects described, the KD usually does not have long-term tolerability, which not just introduces barriers to compliance by caregivers and patients but also compromises the clinical efficacy of the therapy. Although several KD variants are available to increase adherence, additional modifications are still needed and should be specifically designed for distinct diseases to increase both efficacy and tolerability, while facilitating dynamic adjustment to minimize the short- and long-term adverse effects.

The mechanisms of action of the KD, especially at the cellular and molecular levels, in different types of diseases, are poorly understood. As a metabolic therapy, the KD may function via a combination of multiple and complex mechanisms involving metabolic, cellular, and molecular responses. Recent studies have shown that the gut microbiota plays an important role in these processes. Given that the composition of the gut microbiota varies in specific diseases, the possibility of disease-specific mechanisms of KD that are mediated through a functional axis that originates with gut microbes is worth exploring regarding the beneficial effects and side effects of the KD. Therefore, further in-depth investigations into the intrinsic therapeutic mechanisms of the KD in different diseases are needed, as it will not only provide insights into disease pathogenesis from new perspectives, but also lead to the identification of key intermediate biochemical pathways, molecules, and/or other factors, such as gut microbes, that govern the KD treatment-related effects, and these can be utilized as promising targets for drug design or the development of novel customized interventional strategies to mirror the effects of KD.
